# Preparation of Nanocellulose Reinforced Chitosan Films, Cross-Linked by Adipic Acid

**DOI:** 10.3390/ijms18020396

**Published:** 2017-02-13

**Authors:** Pouria Falamarzpour, Tayebeh Behzad, Akram Zamani

**Affiliations:** 1 Department of Chemical Engineering, Isfahan University of Technology, Isfahan 84156-83111, Iran; p.falamarzpour@gmail.com (P.F.); tbehzad@cc.iut.ac.ir (T.B.); 2 Swedish Centre for Resource Recovery, University of Borås, 50190 Borås, Sweden

**Keywords:** acetic acid, adipic acid, chitosan, cross-linking, mechanical properties, nanocomposite film

## Abstract

Adipic acid, an abundant and nontoxic compound, was used to dissolve and cross-link chitosan. After the preparation of chitosan films through casting technique, the in situ amidation reaction was performed at 80–100 °C as verified by Fourier transform infrared (FT-IR). The reaction was accompanied by the release of water which was employed to investigate the reaction kinetics. Accordingly, the reaction rate followed the first-order model and Arrhenius equation, and the activation energy was calculated to be 18 kJ/mol. Furthermore, the mechanical properties of the chitosan films were comprehensively studied. First, optimal curing conditions (84 °C, 93 min) were introduced through a central composite design. In order to evaluate the effects of adipic acid, the mechanical properties of physically cross-linked (uncured), chemically cross-linked (cured), and uncross-linked (prepared by acetic acid) films were compared. The use of adipic acid improved the tensile strength of uncured and chemically cross-linked films more than 60% and 113%, respectively. Finally, the effect of cellulose nanofibrils (CNFs) on the mechanical performance of cured films, in the presence of glycerol as a plasticizer, was investigated. The plasticized chitosan films reinforced by 5 wt % CNFs showed superior properties as a promising material for the development of chitosan-based biomaterials.

## 1. Introduction

Environmental issues, regarding the consumption of petroleum-based products, raise serious efforts to employ alternative materials from natural resources. Nowadays, more attention has been paid to polysaccharides as polymeric renewable materials. This is not only owing to their natural abundance, but also because of their interesting properties and applications. Chitosan is the deacetylated form and the most important derivative of chitin, the second most abundant polysaccharide in nature after cellulose. Chitosan has shown an excellent film-forming ability. High transparency, biodegradability, biocompatibility, antimicrobial activity, and moderate values of water and oxygen permeability are among the superior characteristics of chitosan films which can be utilized for food packaging and coating to prevent contamination and microbial spoilage and, therefore, improve quality and shelf life of food products [1,2,3,4].

Chitosan is soluble in dilute organic acid solutions because of the presence of non-bonding pairs of electrons in the amino groups, which are protonated in acidic solutions. Moreover, gaining the benefit of the strong nucleophilic behavior of these electrons, chitosan reacts with active groups such as aldehyde and ketone [5,6]. In order to prepare the chitosan films, chitosan is usually dissolved in the acetic acid solution and the so-called casting technique is employed to obtain the films [1,2]. If di-functional carboxylic acids, such as succinic acid, glutaric acid, and adipic acid, are employed for dissolution of chitosan, there will be an opportunity for ionic cross-linking between the carboxyl groups of dicarboxylic acids and the amino groups of chitosan chains. These interactions significantly alter the properties of chitosan solutions through physical gelation and formation of a three-dimensional network [7]. Chen et al. [8] used different kinds of organic acids (acetic acid, oxalic acid, succinic acid, malic acid, and adipic acid) to fabricate chitosan membranes. They observed that by replacing acetic acid with dicarboxylic acids, the properties of membranes were significantly improved. Furthermore, they reported that adipic acid, because of its longer carbon backbone, brings more flexibility. Therefore, this acid is more effective than the other carboxylic acids in the improvement of mechanical properties of chitosan films. Similarly, Mitra et al. [9] demonstrated that these interactions significantly improved the mechanical properties and thermal stability of chitosan.

In addition to the ionic interaction, cross-linking can also be carried out through chemical reactions with di-functional agents, such as glutaraldehyde, which leads to the formation of covalent linkages between the chitosan chains [10]. However, cross-linking agents are usually toxic and, therefore, biocompatibility of the resultant biopolymer material is questionable [11]. Interestingly, chitosan films prepared using adipic acid can undergo a chemical amidation reaction at elevated temperatures. This chemical cross-linking reaction can improve the properties of chitosan. Although in situ cross-linking of chitosan with adipic acid has been reported [12], a deep investigation has not been performed on the kinetics of this reaction.

Adipic acid is the most important industrial dicarboxylic acid widely used for the production of nylon 66 and polyurethane [13]. It is a nontoxic and biocompatible compound which has several applications in the food industry, e.g., as a flavorant, acidulating agent, and gelling aid [14]. Recently, biological methods for producing adipic acid from renewable fatty acid feedstocks have been developed [15]. Accordingly, adipic acid can be utilized for the preparation of different chitosan-based biomaterials, especially for biomedical applications, such as drug delivery systems, artificial skin, wound dressing, and tissue engineering, where nontoxicity is an essential aspect [16].

To improve the mechanical properties of chitosan films for practical applications, additives, such as fillers and plasticizers, are needed. Effective plasticizers should have a similar chemical structure to the polymer. Polyols, such as glycerol, which contain hydrophilic groups, are appropriate as plasticizers for chitosan films since chitosan is a hydrophilic biopolymer. Glycerol is the best-known plasticizer of chitosan, and previous studies showed that implementation of 20 wt % glycerol content is sufficient to improve the flexibility of chitosan films [1,17]. Furthermore, numerous investigations studied chitosan films reinforced by cellulose nanofibrils (CNFs). CNFs can be isolated from cellulose resources, such as wood and agricultural crop residues, through a chemo-mechanical process. CNFs are highly crystalline rod-shaped nanomaterials with a high aspect ratio and a large specific surface area. Typical diameters of CNFs are 5–50 nm and fiber lengths can vary in a wide range, from a few hundred nanometers to several micrometers. The CNF extraction process contains physiochemical treatments, including base and acid hydrolysis, and bleaching, followed by high shear mechanical forces, such as high-pressure homogenizers, ultrasonic homogenizers, or grinders which are used to delaminate and separate microfibrils and liberate the nanosized crystalline fibrils [1,2,18]. The similar structure of cellulose and chitosan, and their ability to form hydrogen bonds leads to the formation of a strong interface that is a desirable approach to prepare low-cost, lightweight, and high-performance nanocomposite materials.

In the present study, firstly the reaction kinetics of chitosan-adipic acid was investigated. In the next step, the conditions to optimize the cross-linking degree (CLD %), along with the maximum tensile strength (TS) of the chemically cross-linked chitosan films were established. Then, by revealing the optimal curing conditions, a comprehensive comparison of the mechanical properties of chitosan films, including the films prepared by acetic acid (uncross-linked), adipic acid (physically cross-linked (uncured)), and adipic acid cured with and without plasticizer and CNFs was conducted.

## 2. Results

### 2.1. Fourier Transform Infrared (FT-IR) Analysis to Verify Amide Bond

**Figure 1:**
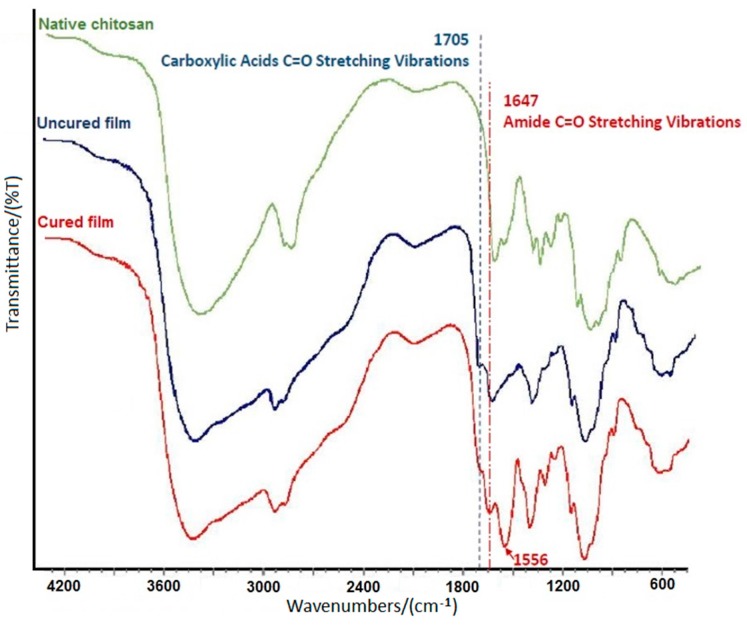
Fourier transform infrared (FT-IR) spectra of native chitosan, uncured chitosan-adipic acid film, and chitosan-adipic acid cured film at 90 °C for 60 min.

To investigate the changes in the chemical structure of chitosan films after cross-linking, Fourier transform infrared (FT-IR) analysis was conducted. The FT-IR spectra of native chitosan and chitosan-adipic acid films (uncured and chemically cross-linked at 90 °C for 60 min, respectively) are shown in Figure 1. The strong and wide peak in the 3500–3300 cm^−1^ zone of the native chitosan spectrum is attributed to the O–H stretching vibration of the hydrogen bond. Additionally, an N–H stretching peak overlaps in the same area. In chitosan films, especially cured ones, this peak becomes wider and sharper, indicating an increase in the number of hydrogen bonds [19]. Compared to native chitosan, the peak appeared at 1705 cm^−1^ in the spectra of chitosan-adipic acid films was assigned to C=O (H-bonded) due to the presence of adipic acid. By converting the carboxyl groups into amide bonds during the cross-linking reaction, the intensity of this peak decreased, as demonstrated in the cured chitosan film spectrum. This suggests the formation of amide bonds and was further confirmed by one of the best amide characteristics. Amides show a very detectable strong C=O peak at 1680–1630 cm^−1^ which is observable in the cured chitosan curve at 1647 cm^−1^. It can be noticed that N–H bending vibrations (at 1556 cm^−1^), which are seen in both primary and secondary amides, partially overlap this peak [20,21]. Furthermore, there is no peak related to the ester C=O stretching vibration at 1750–1735 cm^−1^. In other words, the functional groups of adipic acid did not react with the hydroxyl groups of chitosan [12,20]. Therefore, the chemical cross-linking reaction was only the amidation between the carboxyl groups of adipic acid and the amino groups of chitosan [12].

### 2.2. Kinetics of the Cross-Linking Reaction

**Figure 2:**
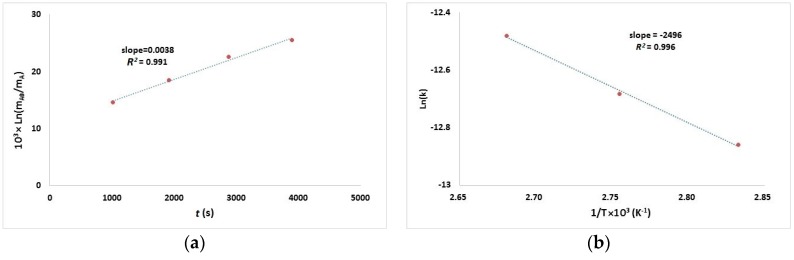
(**a**) The plot of Ln(*m*_A0_/*m*_A_) against time *t* at 100 °C; and (**b**) the relationship between the reaction rate constant and temperature.

The plot of Ln(*m*_A0_/*m*_A_) vs. time for the curing reaction at 100 °C is shown in Figure 2a. According to Equation (3), the linear correlation between Ln(*m*_A0_/*m*_A_) and time is in agreement with the first-order reaction rate model [22,23]. Through the equation of the fitted line, from the intercept, the rate constant at 100 °C was obtained. The same procedure was repeated for the other two temperatures. After obtaining the values of the rate constants, the temperature-dependence behavior of the reaction rate was investigated. Figure 2b shows the plot of Ln(*k*) vs. 1/*T*. The linear relationship between them suggests that the temperature effect follows the Arrhenius equation [22]. Therefore, from the slope of this line, the activation energy (*E*a) was calculated to be about 18 kJ/mol.

### 2.3. Mechanical Properties

#### 2.3.1. Optimization of Chemical Cross-Linking Reaction

**Figure 3:**
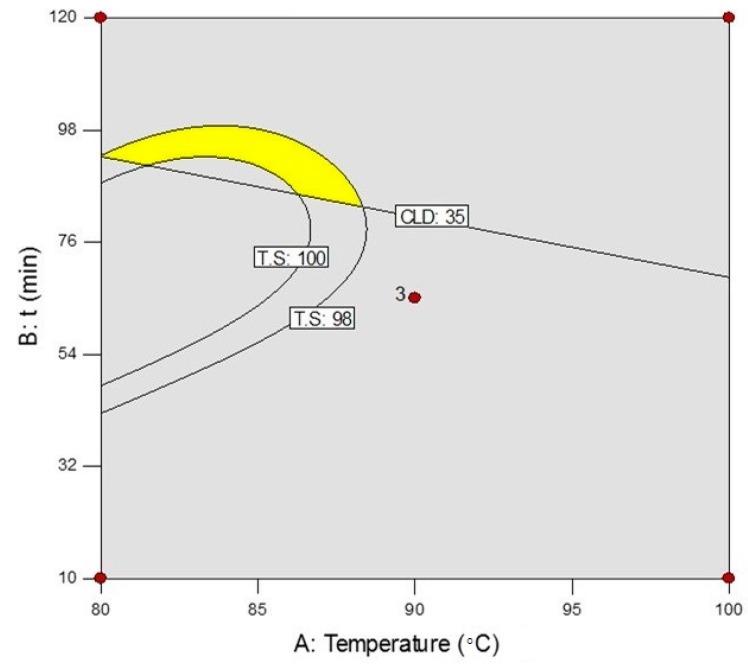
The overlay plot of cross-linking degree (CLD) and Tensile strength (TS). The yellow region is the intersection area of the criteria limits where the optimal conditions were designated. The red point in the middle of this picture shows the condition of the central point i.e., 90 °C, 60 min.

To obtain the correlation between the mechanical strength and the cross-link density of cured chitosan-adipic acid native films (without CNFs and plasticizer), the design of the experiments was performed. Table 1 represents the conditions and experimental responses for each run, which illustrates that the tensile strength (TS) was increased by increasing the CLD % until 30%, while it was decreased at higher CLD levels. Similar behavior was observed for elongation at break (EB %). However, the modulus was increased by increasing the CLD %. A linear model described the CLD % variation based on time and temperature (time interval of 10–120 min and temperature of 80–100 °C). Although CLDs are designed to estimate the responses through a quadratic model, for TS the cubic model illustrates a better behavior (the quadratic model was also statistically significant: *F*-value = 75.16, and *p*-value = 0.0132). All regressions were highly significant (*F*-value = 352.31, and *p*-value < 0.0001 for CLD % and *F*-value = 276.91, and *p*-value < 0.0036 for TS), and also the *R*^2^ coefficients were adequate (0.988 for CLD %, and 0.998 for TS), demonstrating the models are suitable for representing the responses. Figure 3 is the overlay plot for CLD % > 35%, and TS > 98 MPa as criteria. The optimal cross-linking conditions, defined as the yellow area in Figure 3, were 84 °C for 93 min (80 °C and 93 min for the quadratic model). Cross-linking of chitosan films under the optimal conditions improved the mechanical performance, especially TS, which was increased by approximately 30%.

#### 2.3.2. The Effects of Adipic Acid, Cellulose Nanofibrils (CNFs), and Plasticizer on the Mechanical Properties of the Films

To evaluate the effect of the acid type and the addition of CNFs and plasticizer, the mechanical properties of various films were compared and the results are summarized in Table 2. The use of adipic acid (CSAd) improved the TS of chitosan native films more than 60% in comparison with acetic acid film (CSAc for short); however, the flexibility was reduced about 50%. Additionally, by curing adipic acid films at optimal conditions (CScAd), the TS was enhanced by 113% compared to CSAc. In addition, adipic acid increased the Young’s modulus (YM); however, this effect was very impressive when plasticizer was added. As expected, the addition of 20 wt % glycerol as the plasticizer resulted in a reduction of the modulus of plasticized acetic acid–chitosan film (pCSAc) by about 10 times. A similar behavior was observed for adipic acid film (pCSAd); however, the modulus were almost 4.5 times more than that of pCSAc. This value was also enhanced up to six times with the curing of the film (pCScAd) as compared to pCSAc. Glycerol implementation resulted in the enhancement of the EB % of chitosan films up to 280% (pCSAc), 390% (pCSAd), and 220% (pCScAd), respectively; however, the film’s strength significantly decreased, which was very conspicuous for pCSAc (about 47%). This decrease was only 13% for the plasticized uncured adipic acid film (pCSAd) and 21% for the optimally cured one (pCScAd). In other words, plasticized adipic acid uncured film is 165% stronger than the acetic acid film (pCSAc).

Next, the effects of CNFs were investigated. For this purpose 3, 5, and 7 wt % CNFs were added to the chitosan solution. According to Table 2, adding more than 5 wt % CNFs reduced the strength and flexibility of the films; therefore, the optimal percentage of CNFs was considered to be 5 wt % for both films. The addition of 5 wt % CNFs and 20 wt % plasticizer to acetic acid film (p5CSAc) made the strength equal to that of the native film (CSAc), significantly decreased the modulus, and improved the EB % by approximately two times compared to the acetic acid native film. In contrast, all mechanical performances of optimally cured adipic acid film were improved by the addition of 5 wt % CNFs and 20 wt % glycerol (p5CScAd). The TS was increased and reached 127 MPa, the EB % increased approximately two times, and the modulus remained constant.

## 3. Discussion

The development of nontoxic and biocompatible chitosan-based products is important in the biomedical engineering and food industries. Using conventional materials such as acetic acid (as the most common solvent of chitosan) or glutaraldehyde (the well-known chitosan cross-linker) raises several challenges; therefore, new materials and processes need to be developed. Adipic acid is abundant, biocompatible, and nontoxic, and it has the potential to replace both acetic acid and glutaraldehyde in chitosan products.

In this study, a simple and convenient chemical reaction was presented for the cross-linking of chitosan by adipic acid. The amidation reaction between chitosan and carboxylic acids has been reported in several studies. Bodnar et al. [24] prepared chitosan nanoparticles cross-linked with di- and tri-carboxylic acids (succinic acid, malic acid, tartaric acid, and citric acid-1-hydrate). The same reaction was performed at a lower temperature by using a carbodiimide as a condensation agent. Moreover, Valderruten et al. [25] synthesized chitosan hydrogels chemically cross-linked by dicarboxylic acids (adipic, glutaric, and succinic acids) and carboxyl activating agents (*N*-hydroxysuccinimide and 1-ethyl-3-(3-dimethylaminopropyl) carbodiimide). Cai et al. [12] presented a straightforward procedure for the chitosan-adipic acid film formation in situ cross-linking reaction. Accordingly, the carboxylic acid groups of adipic acid and the amino groups of chitosan formed amide bonds merely by heating at 80–100 °C for 40–60 min. The same procedure was followed in this work; however, the achieved kinetics results were not in agreement with Cai et al.’s work.

In this research, the weight loss values due to the condensation reaction were used in order to investigate the kinetics of the curing reaction. The activation energy which was calculated in the kinetics section (*E*a = 18 kJ/mol) is in contrast with the estimated value of the amidation activation energy without the presence of a catalyst, which is roughly 80 kJ/mol (20 kcal/mol) as obtained from previous studies [26]. Additionally, in Cai et al.’s [12] work, the activation energy of the reaction was reported as 60.6 and 76.17 kJ/mol for 40 and 60 min reaction times, respectively. In order to avoid any ambiguity, it must be emphasized that a linear relationship between Ln(*k*) and 1/T, implies that the reaction is in agreement with the Arrhenius equation not the first-order reaction rate model. To investigate the latter, the variation of Ln(*m*_A0_/*m*_A_) versus time at a constant temperature should be linearly plotted. The reaction constant is obtained from the slope. Therefore, it seems impossible to obtain two constants at a constant temperature in this approach. Consequently, obtaining two values for the activation energy is not possible with this method. This lower activation energy in the current study might be attributed to the fact that the formation of ionic bonds between the carboxylic acid groups of adipic acid and the amino groups of chitosan created a platform to facilitate the amidation reaction. In other words, these functional groups were held together by ionic linkages and just a lower energy was required for structural rearrangements and formation of the reaction byproduct.

In this work, a comprehensive study on the mechanical properties of chitosan films was conducted. First, the effect of chemical cross-linking on the mechanical properties of chitosan-adipic acid native films (without CNFs and plasticizer) was investigated. The results showed that the curing reaction may have different effects on the enhancement/reduction of the strength and flexibility of adipic acid–based films. Desired conditions were determined through the design of the experiments. Similar behavior was reported for the effects of chemical cross-linking on the mechanical properties of chitosan and chitosan-based films. Aryaei et al. [27] observed that the cross-linking significantly improved the hardness and elastic modulus of chitosan films. Additionally, results showed that the cross-linking caused more brittle behavior. According to Jin et al. [11], the reduction of the chitosan-to-genipin ratio (or increasing CLD %) in chitosan/poly(ethylene oxide) films would be followed by similar results (TS and EB % were first increased by increasing the CLD %, and then decreased). Next, the effect of the acid type on the mechanical properties of chitosan films was investigated. The use of adipic acid instead of acetic acid had some special advantages. The tensile strength was significantly improved, although the EB % was slightly decreased. Adipic acid prevented excessive loss in the modulus and strength due to the addition of glycerol. In addition, it gave the possibility for chemical cross-linking of chitosan under controllable kinetics and CLD %. Curing at optimal conditions also improved the chitosan properties.

The mechanical properties of the films depend on the inter- and intra-molecular interactions and the chains’ flexibility. Cross-linking (physical as well as chemical) decreases the ability of chitosan chains for slippage, and results in a significant increase in the modulus. The high performance of chitosan-adipic acid films suggests favorable adipic acid–chitosan interactions. Hydrogen bonds and ionic interactions due to the proton exchange between the COOH groups of adipic acid and the NH_2_ groups of chitosan provide the physical cross-linking of chitosan by adipic acid. Mitra et al. [9] demonstrated that these interactions significantly improved the mechanical properties of chitosan. However, excessive CLD % brings restrictions in the molecular motion and flexibility and, consequently, reduces the mechanical performance of the films.

According to the free volume theory, the addition of low-molecular-weight plasticizers increases the intermolecular spaces and the free volume of the polymeric matrix, which results in increasing the molecular mobility and flexibility of the material. Extensive intermolecular forces lead to a brittle behavior. Plasticizers also reduce polymer-polymer interactions (hydrogen bonds here) and form secondary interactions with polymer chains, causing adjacent chains to move apart and decreasing film rigidity and enhancing flexibility. On the other hand, plasticizers decrease the crystallinity of biopolymer films, leading to a significant decrease in the film strength and modulus [1,28,29]. The results obtained in this study are in line with this theory.

Cellulose nanofibrils, as expected, present the inverse effects. More specifically, CNFs improve the strength and stiffness of the films, but impair elongation, as previously reported for chitosan films [1,2,18]. The increased strength and modulus of the nanocomposites suggest good fiber-polymer adhesion interactions. Principally, the mechanical properties of chitosan-based products depend on the interactions between chitosan and the other components of the system, including the cross-linker, plasticizer, and CNFs. The excellent mechanical properties of plasticized chitosan-adipic acid films reinforced by CNFs suggest that the non-covalent interactions play a major role in the high performance of the films. The strength and modulus of the films are higher than the reported values for typical synthetic or biobased polymeric films (Table 3). However, the poor elongation at break of the films, which is the usual drawback of chitosan films, creates some restrictions in applications where high flexibility is required.

## 4. Materials and Methods

### 4.1. Materials

Chitosan (degree of deacetylation: 85%, viscosity of 1 wt % chitosan in 1 wt % acetic acid aqueous solution: 60 mPa·s) and adipic acid (99.5% purity) were purchased from BioLog, Landsberg, Germany and UNI-CHEM, Hangzhou, China, respectively. CNF aqueous suspension (0.95 wt %) was prepared by a chemo-mechanical procedure according to previous research [32]. Double-distilled water was used in this study to prepare solutions. All materials were used without further purification.

### 4.2. Chitosan Film Preparations

To accomplish the kinetics study, chitosan-adipic acid films were prepared. Specified amount of chitosan was added to an adipic acid aqueous solution and mixed for 2 h to obtain a homogeneous solution consisting of chitosan (1 wt %) and adipic acid (0.37 wt %). The Ratio of adipic acid to chitosan (0.37 g/g) was chosen to have the same number of amino groups (of chitosan) and carboxylic acid groups (of adipic acid) in the obtained films. After removal of air bubbles, the solution was cast in 90 mm diameter plastic Petri dishes and placed on a flat surface at ambient temperature for four days. Further drying operation was performed in a vacuum oven for 48 h at 35 °C. Then, heated films were cooled to room temperature in a desiccator for 10 min and the initial weight of uncured films was measured as *m*_A0_. To perform the chemical crosslinking (curing) reaction, dried chitosan films were heated at 80, 90, or 100 °C for 10–120 min in a vacuum oven (90–100 mBar; Memmert, Germany). At these conditions, because of the water release as the byproduct of the amidation reaction [33], a slight weight loss was observed. The secondary weights (*m*_A_) of films were measured after cooling to room temperature in a desiccator for 10 min.

In order to characterize mechanical properties of nanocomposites, 1 g chitosan was dissolved in 100 mL adipic acid (0.37 wt %) or acetic acid (1 wt %) solutions and mixed for 2 h to obtain a homogeneous solution. Then, a specified amount of CNFs suspension and 0.2 g glycerol were added to the solution and mixed under vigorous magnetic stirring at room temperature for 5 h. Finally, the mixture was homogenized by ultrasonication in a water bath for 10 min to achieve a uniform dispersion. All of the solutions were cast in the Petri dishes and air-dried for 6 days.

### 4.3. Kinetics Study

To study the kinetics of the cross-linking reaction, the first-order kinetics reaction and Arrhenius equation were investigated. The carboxyl group of adipic acid ionically linked to the amino group of chitosan was considered as reactant A. The reaction took place and products, including the amide bond (B) and H_2_O molecule (C), were formed. According to the first-order reaction model [22,23]:

−*r*_A_ = −d*C*_A_/d*t* = *kC*_A_ (1)

where *r*_A_, *C*_A_, *t*, and, *k* represent the reaction rate, concentration of A, time, and rate constant, respectively. Integration of Equation (1) at constant a temperature between the limits of *C*_A0_ at *t* = 0, and *C*_A_ at *t* gives:

Ln(*C*_A0_/*C*_A_) = *kt* + *c* (2)

where *c* is a constant value.

The volume of the sample was assumed to be constant. By using the definition of concentration (*C*_A_ = *m*_A_/*V*) and replacement in Equation (2), Equation (3) can be derived as follow:

Ln[(*m*_A0_/*V*)/(*m*_A_/*V*)] = Ln(*m*_A0_/*m*_A_) = *kt* + *c* (3)

where *m*_A0_ and *m*_A_ represent the initial weights of the film and the weight of the film at time *t*, respectively. The weight of water which was produced in the reaction is obtained as (*m*_A0_ − *m*_A_).

Equation (3) shows that, for a first-order reaction, plotting Ln(*m*_A0_/*m*_A_) vs. *t* gives a straight line with a slope of *k*. For each temperature (80, 90, and 100 °C) at least three points with three different times were investigated.

The temperature dependence of the reaction rate constant of many reactions is described by the Arrhenius equation [22]:

Ln(*k*) = Ln(*A*) − *E*a/*RT* (4)

where *E*a and *A* represent the activation energy and the frequency factor, respectively. For these reactions, plotting of Ln(*k*) against 1/*T* gives a straight line with slope of −*E*a/*R*.

The cross-linking degree (CLD) is defined as the ratio of the produced H_2_O (the weight loss of film) to its theoretical value (Δ*m*_th_), which is calculated based on the degree of deacetylation (Equation (5)):

CLD % = (*m*_A0_ − *m*_A_)/∆*m*_th_ × 100 (5)

### 4.4. Film Characterization

#### 4.4.1. Fourier Transform Infrared (FT-IR) Analysis

FT-IR analysis was conducted using an FT-IR spectrophotometer (WQF-510, Beijing Rayleigh Analytical Instrument Corporation, Beijing, China). Small pieces of film and dried potassium bromide (KBr) were thoroughly ground to form pellets. Spectra were recorded with a resolution of 4 cm^−1^ with a total of 32 scans, in the range 4400–400 cm^−1^.

#### 4.4.2. Mechanical Properties

Film thicknesses were measured with a constant-load micrometer. Mechanical properties of samples, i.e., tensile strength (TS), elongation at break (EB), and Young’s modulus (YM), were analyzed according to ASTM D882 using the Zwick Universal Testing Machine—1446-60, Ulm, Germany. Rectangular pieces of films (45 × 15 mm) were extended by steel grips at the rate of 10 mm/min and gauge length of 30 mm. Before test, the samples were stored in plastic bags at ambient conditions for one week. Each test was repeated at least three times at room temperature. The results were analyzed by Design Expert 9 (Stat-Ease, Inc., Minneapolis, MN, USA). A design of the experiment based on a rotatable central composite design (CCD) was established to optimize curing conditions by assessing CLD % and TS of films. The models developed to describe the responses were evaluated in terms of *F*-value, *p*-value, and *R*^2^ coefficient. To determine optimal experimental conditions that could provide maximum values of TS and desired CLD %, the “overlay plot” was used. The criterion was the maximum actual tensile strength at CLD above 35%.

## 5. Conclusions

The present study developed a novel approach to prepare chitosan films. Adipic acid, as an abundant, nontoxic, and biologically compatible solvent, was used to cross-link chitosan through both ionic interactions and covalent amide bonds. Furthermore, the mechanical properties of chitosan films were improved by the addition of 20 wt % glycerol and 5 wt % CNFs. The use of adipic acid in this step possessed an advantage: it prevented the loss of strength and modulus by forming an ionic interaction with the chitosan and cross-linking the chains. The films possessed a high strength and modulus. The biological production and biocompatibility of both chitosan and adipic acids are the most important promising features of chitosan-adipic acid films which provide the opportunity for the application of the obtained films in the food packaging and medical industries.

## Figures and Tables

**Table 1:** Tensile strength (TS), cross-linking degree (CLD), elongation at break (EB), and Young’s modulus (YM) of chitosan films prepared at different curing times and temperatures.

Run	Factor 1: Temperature (°C)	Factor 2: Time (min)	Response 1: TS (MPa)	Response 2: CLD (%)	EB (%)	YM (MPa)
1	35	0	78.14	0	3.79	4798
2	80	10	76.07	14.4	4.06	5014
3	100	10	79.61	18.5	4.78	5038
4	75	65	100.18	27.7	4.22	5115
5	90	65	95.76	30.9	3.81	5194
6	90 ^a^	65	93.14	30.9	3.76	5189
7	90	65	96.69	30.9	3.93	5208
8	104	65	92.27	34.8	3.39	5237
9	80	120	80.97	40.5	3.00	5356
10	90	143	62.84	48.6	1.73	5569
11	100	120	52.52	51.6	1.42	5775

^a^ Central point: 90 °C, 60 min.

**Table 2:** Comparison of mechanical properties of chitosan films.

Chitosan Film	Solvent	CNFs (gr/gr CS)	Glycerol (gr/gr CS)	Curing	TS (MPa)	EB (%)	YM (MPa)
CSAc	Ac	0	0	No	48.45	8.11	3183
CSAd	Ad	0	0	No	78.14	3.79	4798
CScAd	Ad	0	0	Yes	103.25	4.37	5434
pCSAc	Ac	0	0.2	No	25.69	31.03	381
pCSAd	Ad	0	0.2	No	68.32	18.61	1736
pCScAd	Ad	0	0.2	Yes	81.57	13.98	2297
p3CSAc	Ac	0.03	0.2	No	38.18	24.73	728
p3CScAd	Ad	0.03	0.2	Yes	113.41	12.55	3004
p5CSAc	Ac	0.05	0.2	No	45.66	21.40	983
p5CScAd	Ad	0.05	0.2	Yes	127.84	11.93	4715
p7CSAc	Ac	0.07	0.2	No	40.03	17.89	1027
p7CScAd	Ad	0.07	0.2	Yes	109.37	8.51	4082

p: plasticized; 3, 5, and 7: CNF content; CSAc: chitosan-acetic acid film; CSAd: chitosan-adipic acid film (uncured); CScAd: chitosan-optimal cured adipic acid film.

**Table 3:** Comparison of mechanical properties of chitosan films with several polymers.

Materials	TS (MPa)	EB (%)	YM (MPa)
p5CScAd	127	11.93	4715
CSAc1	55–62	4.58	-
CSAc2	79	8.58	1590
5CSAc	99	3.98	2971
p15CSAc	52.7	10.3	1368
CS-GA	25	19.8	-
Alginate	18–49	6.5-13	122–480
Gelatin	47–85	3-8	1978–2245
LDPE	8–31	125–675	200–500
PP	31–43	100–600	1140–1550
PS	14–70	1.0–2.3	2280–3280
PVC	10–55	200–450	3–21

p5CscAA: 20 wt % glycerol, 15 wt % CNFs, chitosan-adipic acid film cross-linked at 84 °C for 93 min (this work); CSAc1: chitosan-acetic acid film [30]; CSAc2: chitosan-acetic acid film [2]; 5CSAc: 5 wt % cellulose nanofibers, chitosan-acetic acid film [2]; p15CSAc: 18 wt % glycerol, 15 wt % cellulose nanofibers, chitosan-acetic acid film [1]; CS-GA: chitosan film cross-linked by glutaraldehyde [31]; LDPE: low-density polyethylene; PP: polypropylene; PS: polystyrene; PVC: poly(vinyl chloride) [1].

## Abbreviations

CS	Chitosan
Ac	Acetic acid
Ad	Adipic acid
CNFs	Cellulose nanofibrils
CLD	Cross-linking degree
TS	Tensile strength
EB	Elongation at break
YM	Young’s modulus
CSAd	Chitosan-adipic acid uncured film
CSAc	Chitosan-acetic acid film
CScAd	Chitosan-adipic acid chemically cross-linked film
pCSAc	20 wt % glycerol plasticized chitosan-acetic acid film
pCScAd	20 wt % glycerol plasticized chitosan-adipic acid chemically cross-linked film
P3CSAc	Plasticized chitosan-acetic acid film reinforced by 3 wt % CNFs
p5CScAd	Plasticized chitosan-adipic acid chemically cross-linked film reinforced by 5 wt % CNFs
